# Commencements

**Published:** 1894-05

**Authors:** 


### COMMENCEMENTS.

### American College of Dental Surgery.

The eighth annual commencement exercises of the American College of Dental Surgery were held at the Grand Opera House, Chicago, Ills., on Monday, April 2, 1894, at 2:30 o'clock.

The faculty address was delivered by Dr. Edward H. Angle, and the valedictory, "The Elements of Success," by Dr. Alice Menges.

The degree of D.D.S. was conferred on the following graduates by Dr. B. J. Cigrand, President of the Board of Directors:

E. D. Allison.

E. Aiston.

E. H. Bulson.

L. A. Beyer.

C. W. Barlow.

C. W. Blackburn.

Alden Carpenter.

L. A. Colestock.

D. H. Davis.

F. E. Downs.

C. L. Eberts.

Peter Frey.

E. B. Griffin.

S. A. Henry.

Ida N. Harter.

A. G. Hayden.

L. A. Kelley.

E. L. Ketcham.

I. B. Locke.

F. Leggo.

S. E. Lane.

Alice Menges.

A. H. Murdow.

J. F. Mensel.

G. W. Menges.

W. J. Morgan.

J. C. Monzon.

J. C. McDermott.

C. E. Neptune.

B. F. Nichols.

J. T. Perrault.

F. E. Roach.

E. A. Randall.

C. Scheer.

D. E. Smith.

T. M. Smith.

J. Senty.

F. Senty.

H. S. Stevenson.

W. R. Toye.

J. D. VanVleck.

D. M. Willard.

G. W. Wyatt.

Total, 43.

### Royal College of Dental Surgeons of Ontario.

The number of matriculates for the session of 1893-94, at the Royal College of Dental Surgeons of Ontario, Toronto, Canada, was ninety-eight.

No formal commencement was held, but the following students passed the final examination and received the title of L.D.S., and a certificate of license to practice dentistry in Ontario:

Alton Anderson.

C. N. Abbott.

J. W. Bell.

F. E. Beemer.

J. D. Cameron.

M. F. Cross.

Charles Cobban.

Charles Colter.

Donald Davidson.

G. A. Dewar.

W. R. Greene.

H. A. Galloway.

F. G. Hughes.

V. H. Lyon.

A. A. McKenzie.

J. R. Mitchell.

D. Marshall.

J. McKnight.

A. E. McCordick.

G. A. Newton.

B. F. Nicholls.

G. R. Patterson.

R. J. Read.

W. A. Scott.

E. A. Totten.

H. P. Thompson.

W. T. Wood.

F. L. Wood.

A. E. Webster.

All of the Province of Ontario.

Total, 29.

### Ohio College of Dental Surgery.

The forty-eighth annual commencement of the Ohio College of Dental Surgery, Department of Dentistry, University of Cincinnati, was held at the Odeon, Cincinnati, on Tuesday evening, March 27, 1894, at eight o'clock.

The number of matriculates for the session was one hundred and fifty-one.

An address on the "Winning Qualities in Professional Life," was delivered by Rev. Dr. John W. Simpson, President of Marietta College, and the Class Oration by William C. Elifritz, of Springfield, Ohio.

The degree of D.D.S. was conferred on the following persons by F. A. Hunter, D.D.S., of the Board of Trustees.

| NAME. | RESIDENCE. |
|---|---|
| Charles Verey Alsop | Kentucky |
| John Allen Auld | Michigan |
| Edward Grove Barnett | Ohio |
| Charlie Frederick Bauer | Ohio |
| Fred. Ward Brammer | Ohio |
| Daniel Harrison Collette | Ohio |
| Samuel Robert Collier | Kentucky |
| Frank R. Cunningham | California |
| Will DeZoete | Michigan |
| William Charles Elifritz | Ohio |
| Will A. Gregg | Ohio |
| Charles L. Guthrie | Ohio |
| Horace Greeley Hamilton | Ohio |
| George Robert Jenkins | West Virginia |
| Champin Frank Lauderdale | Ohio |
| Lewis Edwin LePage | Ohio |
| William Frederick Lohaas | Ohio |
| Josiah Mann | New York |
| Austin Freeland Merrell | Maine |
| William Franklin Peebles | Ohio |
| Guy Ballard Saxton | Kansas |
| Herman Schwartz | Ohio |
| John Marshall Scott | Ohio |
| David Andrew Smith | Ohio |
| Richard Storey | Minnesota |
| William Jelley Sullivan | Indiana |
| Edward M. Talbot | Iowa |
| Arthur Lincoln Teeters | Ohio |
| Henry Champlain Taylor | Ohio |
| Lawrence Paul Vandervoort | Ohio |
| Thaddeus Selby Ward | Ohio |
| Will Lewis Whipple | Minnesota |
| T. Irving Way | Ohio |
| Herman Henry Zumstein | Ohio |

Total, 34.

### Indiana Dental College.

The fifteenth annual commencement of the Indiana Dental College was held at the Y. M. C. A. Hall, Indianapolis, on Friday evening, March 23, 1894, at eight o'clock.

The number of matriculates for the session was one hundred and four.

The degree of D.D.S. was conferred on the following graduates:

| NAME. | RESIDENCE. |
|---|---|
| DeVolney Bower | Indiana |
| A. E. Boyce | Illinois |
| L. W. Dailey | Indiana |
| J. D. Gage | South Dakota |
| L. Gonzales | Indiana |
| F. M. Hindman | Illinois |
| D. A. House | Ohio |
| A. F. Hubbard. | Ohio |
| W. E. Hutchinson | Indiana |
| F. A. Lange | Indiana |
| Nannie Margason | Illinois |
| P. R. McNeille | New York |
| Maud Neff | Indiana |
| D. H. Oliver, jr | Indiana |
| T. W. Potter | Indiana |
| R. J. Russell | Indiana |
| J. D. Seibert | Michigan |
| S. N. Sellers | Indiana |
| W. L. Spates | California |
| G. J. Staggs | Indiana |
| F. C. Todd | Indiana |
| Charles Whitted. | Indiana |
| I. M. Whittenburg | Illinois |

Total, 23.

### Baltimore College of Dental Surgery.

The fifty-fourth annual commencement exercises of the Baltimore College of Dental Surgery were held at Albaugh's Lyceum Theatre, on Tuesday evening, March 20, 1894, at 8 o'clock.

The annual oration was delivered by the Rev. R. H. Payne, and the valedictory by E. Hoffmeister, Ph.D.

The degree of D.D.S. was conferred on the following graduates by Prof. R. B. Winder, M.D., D.D.S., Dean of the college:

| NAME. | RESIDENCE. |
|---|---|
| Bernard Bar | Maryland |
| Thomas Edw. Black. | N. Carolina |
| Ellery Percival Blanchard | Maine |
| Emmet Jos. Collins | Georgia |
| Robt. Sinclair Corse, jr | Maryland |
| Jas. Horton Cowen | New York |
| Fred'k North Damon | Massachusetts |
| Wm. Preston Davis | Mississippi |
| Caleb Dorsey | Maryland |
| Adelard Jos. Fortier | Rhode Island |
| Wilbert Ennis Foster | Texas |
| Archie Russell Gardner | Montana |
| Thos. Mauck Guier | Maryland |
| Byron Foster Hadley. | Massachusetts |
| Alfred Chas. Hafke, D.D.S. | Germany |
| Wm. Sprigg Hamilton | Georgia |
| Fred'k England Hawkesworth. | Canada |
| Edw. Hoffmeister, Ph. D. | Maryland |
| Chas. Hutchinson | Virginia |
| Arthur Jay Kiser | Ohio |
| Louis Klingelhoffer | Ohio |
| G. Herman Kopperl | Texas |
| Chas. Bruce McCue | Maryland |
| Wm. Gaston Mizell, jr. | North Carolina |
| Almus Owen | Texas |
| David Burdett Payne. | New York |
| Elton Perry | Texas |
| Wm. Lewis Peters | Massachusetts |
| David Saunders Ray | North Carolina |
| Nicholas Edgar Rierson | N. Carolina |
| Jno. Geo. Schmetzer | South Carolina |
| Geo. Ezra Shattuck | Mississippi |
| Winston Stephens | Florida |
| Geo. Everett Thweatt | Virginia |
| Chas. Richard Twilley | Maryland |
| Wm. Fred'k Walz. | Virginia |
| Dan'l Bitner Williams | Pennsylvania |

Total, 37.

### New York College of Dentistry.

The twenty-eighth annual commencement exercises of the New York College of Dentistry were held in Chickering Hall, New York City, on Tuesday evening, March 13, 1894.

The address to the graduates was delivered by Rev. Percy Stickney Grant, and the valedictory by William G. Beecroft, D.D.S.

The number of matriculates for the session was two hundred and ninety-four.

The degree of D.D.S. was conferred on the following graduates by Professor Frank Abbott, M.D., Dean of the Faculty:

George N. Appleton.

Herbert R. Armstrong.

Charles F. Ash

William G. Beecroft.

Harry M. Bartlett.

James H. Blasdell, jr.

John M. Byers.

Walter H. Colburn.

Louis Cancino, jr.

Thomas C. Cobb.

Percy E. Dowe.

Julius Feiner.

Benjamin Freeman.

Raoul A. Frechette.

Henry Green.

Frederick R. Goddard.

John M. Gillett, M.D.

William C. Gaerste.

Frederick J. Gottlieb.

Charles F. Halpine.

William H. Hopkins.

Marcus C. Hankinson.

Louis H. Hansen.

Joseph H. Holmes.

Oscar A. Krone.

Frank A. Kendall.

Harry O. Logue.

Abram G. Lansing.

James McKenzie, jr.

Timothy H. Murray.

Charles E. Moore.

Vincent P. McLaughlin.

Harry P. Marshall.

Ernest W. Nickerson.

Joseph G. Pease.

Arthur L. Pearson.

William B. Powell.

Joseph Refsum.

Reuben T. Root.

James P. Ruyl.

William H. Rogers.

Joseph E. Smith.

Walter S. Smith.

A. T. Sitterley, jr.

Edward A. Sanford.

David A. Sniflen.

Thomas F. Sweeney.

August Stubenrauch, jr.

Samuel J. Sheckter.

Wilhelm P. Schorn.

Pedro M. Serrano.

Arthur Shorrock.

William A. Swaine.

Albert W. Twiggar.

Timothy C. Tiffany.

Frederick C. Turner.

Hubertus E. Voss.

Edgar E. Woisard.

George W. Watson.

Gerald R. Webster.

Harry A. Woodward.

William Zachman.

Total, 62.

### Philadelphia Dental College.

The thirty-first annual commencement exercises of the Philadelphia Dental College were held in the Chestnut Street Opera House, Philadelphia, Pa., on Thursday, March 8, 1894, at 12 o'clock M.

The number of matriculates for the session was two hundred and ninety-nine.

The address to the graduates was delivered by Prof. S. B. Howell, M.D., D.D.S., and the valedictory by Samuel H. Miller, D.D.S.

The degree of D.D.S. was conferred on the following graduates:

| NAME. | RESIDENCE. | NAME. | RESIDENCE. |
|---|---|---|---|
| John C. Amig | Pennsylvania | W. B. Lapham | New York |
| John W. Anderson | Pennsylvania | Will H. Lawrence. | California |
| Harry T. Avery | Pennsylvania | M. J. Lossing | Canada |
| Willard H. Barnes | New York | C. R. McDermott | Massachusetts |
| Nils Berg | Sweden | T. F. Marovici | Roumania |
| Horace H. Bigelow | Nova Scotia | J. T. Marsden | Pennsylvania |
| Godwin M. Brown | North Carolina | Wm. Mason | Canada |
| W. A. Burns, L.D.S., | Canada | Edwin S. Mershon | Pennsylvania |
| Nellie M. Carle | Pennsylvania | S. H. Miller | Pennsylvania |
| M. J. Cogan | Canada | Robert Milligan | Pennsylvania |
| R. W. Collins | Canada | H. P. Moody | British Columbia |
| S. E. A. Daniel | West Indies | Norman H. Myers | Pennsylvania |
| F. E. Davis | Iowa | Homer Palmour | Georgia |
| Robert Lent Davis | Rhode Island | Geo. B. Parker | New York |
| T. H. Dawes | New Jersey | A. E. Preston | New York |
| W. J. Devereaux | Massachusetts | A. D. Quick | New York |
| A. B. Dorland | Michigan | J. Maude Rankin | New York |
| Fred N. Duncan | New Jersey | L. H. Reinhart | Pennsylvania |
| J. J. Eckel | Pennsylvania | Chas. F. Rodbard | Pennsylvania |
| G. W. Filbert, jr., B. S | Pennsylvania | M. E. Rummel | Ohio |
| W. S. Flower | Pennsylvania | M. P. Shoemaker | New Jersey |
| A. J. Foret | Louisiana | W. D. B. Spry | Canada |
| H. H. Forsythe | Canada | C. H. Steele | Texas |
| T. E. Fouser | Illinois | Roger Steib | Louisiana |
| T. W. Gabel | Pennsylvania | J. E. Stevenson | Wisconsin |
| Percy D. Greene | New York | W. F. Sullivan | Alabama |
| E. Greenwood | England | W. A. Taft | New York |
| D. J. Griswold | Illinois | Chas M. Taylor | Pennsylvania |
| N. C. Heaton | Pennsylvania | Otto Tellefsen | Norway |
| Lynn Himel | Louisiana | J. W. Thatcher | Utah |
| A. Hinman, jr | Oregon | G. L. D. Tompkins | New Jersey |
| O. C. Hole | Illinois | J. F. Thomas | Pennsylvania |
| Will Holloway. | Ohio | D. S. Wells | Vermont |
| Frank M. Howard | Pennsylvania | C. E. Wettlaufer | Canada |
| A. A. Humber | British Columbia | Fred. C. Whiting | France |
| P. A. Hymel | Louisiana | H. Putnam Wisely | Canada |
| H. G. Jaquess | Illinois | S. E. Worster | Kansas |
| A. L. Jenkins | Pennsylvania | Martin Zobel | India |
| Chas. E. King | New Jersey | | Total, 77. |

### University of Maryland—Department of Dental Surgery.

The annual commencement exercises of the Department of Dental Surgery of the University of Maryland were held at the Lyceum Theatre, Baltimore, Friday, March 30, 1894.

The mandamus was read by the Dean, Prof. Ferdinand J. S. Gorgas, M.D., D.D.S.; the address to the graduates was delivered by Rev. Dr. L. T. Townsend, and the class oration by C. Chandler George, Illinois.

The degree of D.D.S. was conferred on the following graduates by Hon. S. Teackle Wallis, LL.D., Provost of the University:

| NAME. | RESIDENCE. | NAME. | RESIDENCE. |
|---|---|---|---|
| Frank E. Blakeslee | New York | William P. Lacy | Virginia |
| William M. H. Burfeind | California | William S. Lindsay | South Carolina |
| James C. Chisholm | Alabama | Harter W. March | Ohio |
| Henry Clay Daniel | North Carolina | Charles A. Matthews | Indiana |
| Harry M. Dommett | New York | James T. Montgomery | South Carolina |
| Charles Augustus Davis | Wisconsin | Redorick Morgan | North Carolina |
| Stanton D. Diffenderfer | Pennsylvania | Leander B. Milbourne | Maryland |
| Thomas Dotterer | South Carolina | James A. Proctor | Virginia |
| Baylis D. Earle | South Carolina | William H. Renoe | Missouri |
| Milton S. Fleming | Louisiana | Clarence P. Reynolds | New York |
| H. Eugene Gee | South Carolina | C. Charles Stanley | South Carolina |
| C. Chandler George | Illinois | Norman R. Smithers | Maryland |
| Edward H. Goldberg | Maryland | Fred. B. Smith | Pennsylvania |
| William J. Hartnett | Connecticut | Richard Channing Walden | Virginia |
| John E. M. Heffelbower | Texas | John Eldridge Walton | Georgia |
| Phineas E. Horton | North Carolina | James Watson | Illinois |
| Ambrose H. Huffman | West Virginia | J. Brenton Wise | South Carolina |

Total, 34.

### Cincinnati College of Dental Surgery.

The third annual commencement of the Cincinnati College of Dental Surgery was held at the Sinton Hall, Y. M. C. A. Building, Cincinnati, O., on Monday evening, April 2, 1894, at eight o'clock.

The opening remarks were made by the Dean of the College, and the valedictory address was delivered by Prof. A. V. Phelps, M.D.

The degree of D.D.S. was conferred upon the following graduates by the Dean, G. S. Junkerman, M.D., D.D.S.:

| | |
|---|---|
| D. R. Anderson. | Wm. P. Murray. |
| Chas. E. Apple. | John F. Zinselmeier. |
| H. R. Hammond. | Total, 5. |

### Meharry Medical College—Dental Department.

The eighth annual commencement exercises of the Meharry Dental Department of Central Tennessee College, Nashville, Tenn., were held at the Gospel Tabernacle, on Thursday evening, February 8, 1894, in connection with those of the Medical and Pharmaceutical Departments.

The number of matriculates for the session was ten.

The address to the graduating classes was delivered by Bishop J. M. Walden, of Cincinnati, Ohio.

The degree of D.D.S. was conferred on the following graduates by the President, Rev. John Braden, D.D.:

| NAME. | RESIDENCE. | NAME. | RESIDENCE. |
|---|---|---|---|
| M. C. Cooper | Texas | A. F. White | Florida |
| G. W. Miller | Tennessee | | |

Total, 3.

### Kansas City Dental College.

The twelfth annual commencement of the Kansas City Dental College was held at Grand Avenue M. E. Church, Kansas City, Missouri, March 5, 1894.

The faculty address was delivered by Alton H. Thompson, D.D.S., Topeka, Kansas, and the valedictory by J. B. Brady, of the graduating class.

The number of matriculates for the session was one hundred and thirteen.

The degree of D.D.S. was conferred on the following graduates by the President of the Faculty, C. B. Hewitt, D.D.S.:

| NAME. | RESIDENCE. | NAME. | RESIDENCE. |
|---|---|---|---|
| Eugene Wesley Allendorph | Kansas | Richard Andrew Millen | Missouri |
| Chas. Canning Allen | Kansas | Schuyler Waller Pile | Kansas |
| James Burns Brady | New Mexico | Chas. Nelson Rathbun | Iowa |
| Harry Evert Duncan | Kansas | Geo. Thomas Reily | Missouri |
| John Hockaday Galliher | Missouri | Ellsworth L. Rothrock | Kansas |
| Henry A. Kinnan | Iowa | Archibald Benj. Stote | Kansas |
| Joseph Edgar McKee | Missouri | Abram Thompson | Kansas |
| James Arthur Miller | California | Augustus Morton Wilkes | Kansas |

Total, 16.

### Ohio Medical University—Department of Dentistry.

The second annual commencement of the Dental Department of the Ohio Medical University was held in connection with those of the departments of Medicine and Pharmacy in the Board of Trade Auditorium, Columbus, Ohio, March 13, 1894.

The number of matriculates for the session was twenty-three.

The degree of D.D.S. was conferred on the following graduates:

| NAME. | RESIDENCE. | NAME. | RESIDENCE. |
|---|---|---|---|
| J. S. Elder | Ohio | James Whiting | Canada |

Total, 2.

### Western Reserve University—Dental Department.

The first annual commencement exercises of the Dental Department of the Western Reserve University were held February 28, 1894, in the Euclid Avenue Presbyterian Church at Cleveland, Ohio, in connection with the Medical Department, which on that day celebrated its fiftieth anniversary.

Prof. William H. Welch, of Johns Hopkins University, delivered the address; subject "Higher Professional Education."

The number of matriculates for the session was thirty.

The degree of D.D.S. was conferred upon the following graduates by Charles F. Thwing, D.D., President of the University:

| NAME. | RESIDENCE. | NAME. | RESIDENCE. |
|---|---|---|---|
| Carl. A. H. Anderson, M.D | Ohio | J. Frank H. Riggs | Pennsylvania |
| Hugh B. Mitchell | Ohio | G. Otis D'Urfae | Ohio |

Total, 4.

### University of Iowa—Dental Department.

The twelfth annual commencement of the Dental Department of the State University of Iowa was held at the Opera House, Iowa City, Thursday, March 15, 1894, at 7:30 P. M.

The number of matriculates for the session was one hundred and forty-eight.

The annual address was delivered by H. E. Downer, A.B., of Davenport, Iowa.

The degree of D.D.S. was conferred on the following graduates by Charles A. Schaeffer, Ph.D., President of the University:

| NAME. | RESIDENCE. | NAME. | RESIDENCE. |
|---|---|---|---|
| G. S. Archer | Iowa | S. L. Ingham | Iowa |
| Maude Baldwin | Wisconsin | W. B. Jayne | Iowa |
| R. W. Baldwin | Wisconsin | H. J. Joy | Iowa |
| O. H. Bemis | Minnesota | W. D. McCabe | Iowa |
| W. G. Bernard | Washington | B. H. McKeeby | Iowa |
| Mallie R. Bowman | Iowa | F. E. Miller | Iowa |
| H. H. Belding | Iowa | E. V. Marsh | South Dakota |
| Chas. W. Bruner | Iowa | W. W. Orebaugh | Iowa |
| M. Clark | Illinois | W. A. Pegg | Pennsylvania |
| H. Daily | Nebraska | J. B. Pherrin | Iowa |
| S. J. Evans | Iowa | M. C. Reno | Iowa |
| E. H. Hollister | Iowa | J. E. Rose | Iowa |
| A. A. Harris | Iowa | J. I. Thompson | Iowa |
| Gard Hicks | Iowa | G. A. Vawter | Illinois |
| L. H. Hinkley | Iowa | W. W. Wold | Minnesota |
| Adel Homer | Iowa | | |

Total, 31.

### The Cleveland University of Medicine and Surgery—Dental Department.

The third annual commencement of the Dental Department of the Cleveland University of Medicine and Surgery was held in connection with that of the Medical Department on Tuesday, March 20, 1894, at Cleveland, O.

The annual address was delivered by Rev. Dr. Levi T. Gilbert.

The degree of D.D.S. was conferred upon the following graduates, viz.:

| NAME. | RESIDENCE. | NAME. | RESIDENCE. |
|---|---|---|---|
| J. G. Colton | Ohio | Fred. A. Herrick | Pennsylvania |
| M. E. Fenton | Ohio | W. G. Johnson | Ohio |

Total, 4.

### Pennsylvania College of Dental Surgery.

The thirty-eighth annual commencement exercises of the Pennsylvania College of Dental Surgery were held at the Academy of Music, Philadelphia, Pa., on Thursday, March 8, 1894, at 12 o'clock M.

The annual address was delivered by Professor Henry Leffman, M.D., D.D.S.

The number of matriculates for the session was two hundred and seventy-three.

The degree of D.D.S. was conferred on the following graduates by I. Minis Hays, M.D., President of the Board of Corporators:

| NAME. | RESIDENCE. | NAME. | RESIDENCE. |
|---|---|---|---|
| F. R. Adams | New York | H. B. Littell | Pennsylvania |
| Martha B. Allaire | New York | James L. Lord | Pennsylvania |
| R. D. Ambrose | Pennsylvania | Vedder Marcellus | New Jersey |
| J. S. Anderson | Canada | W. B. Martin | Pennsylvania |
| H. W. Bender | Pennsylvania | J. R. Megraw | Missouri |
| Carl Bischof | Germany | A. B. Miller | Pennsylvania |
| C. W. Boyer | Pennsylvania | L. M. Monroe | Pennsylvania |
| Louis Britton | Pennsylvania | H. W. Monroe. | Pennsylvania |
| A. G. Brown | New York | W. T. Morton, M.D | Pennsylvania |
| H. W. Burchell | Canada | G. B. Murray | Pennsylvania |
| E. E. Clark | Pennsylvania | Harvey McAdam | Canada |
| Alex. Cornish | Pennsylvania | Eugene Pettit | Pennsylvania |
| B. A. Feltwell | Pennsylvania | E. L. Pitcher | New York |
| L. H. Fisk | Connecticut | Rudolph Probst | Switzerland |
| R. E. Forster | Pennsylvania | H. W. Reid | New York |
| L. D. Fisher | Louisiana | F. Dell Rhodes | Ohio |
| L. McC. Gibbs | Virginia | R. H. Savage | Pennsylvania |
| W. A. Green | Maryland | H. C. Stout | Pennsylvania |
| F. L. Grier | Delaware | C. B. Schupack | Pennsylvania |
| F. A. Hall | Minnesota | Geo. A. Slick | Pennsylvania |
| F. H. Hallen | England | J. W. Story | Pennsylvania |
| Theo. R. Harvey | Pennsylvania | Paul Scheidt | Germany |
| H. E. Hager | Pennsylvania | J. Alberto del Solar | Chili |
| Laura K. Hafer | Pennsylvania | E. A. Thayer | Tennessee |
| J. G. Harter | Pennsylvania | G. W. Troth | Pennsylvania |
| W. S. Hess | Pennsylvania | C. J. Van Horn | Pennsylvania |
| E. R. Hearn | Pennsylvania | J. Wells Van Valzah | Pennsylvania |
| Marie J. Hyndman | Pennsylvania | A. M. Waas | Pennsylvania |
| A. J. Irwin | Canada | Alex. Watson | Canada |
| A. W. Johnson | New York | Ira G. Wells | New York |
| George C. Knox | Pennsylvania | B. B. Williamson | New York |

Total, 62.

### Atlanta Dental College.

The first annual commencement exercises of the Atlanta Dental College were held at Atlanta, Ga., on Monday evening, March 5, 1894.

The number of matriculates for the session was one hundred and eighteen.

The valedictory address was delivered by Mortimer Tuttle, D.D.S., and the annual oration by Mr. James W. Austin.

The degree of D.D.S. was conferred on the following graduates by Judge Wm. R. Hammond, President of the Board of Trustees:

| NAME. | RESIDENCE. | NAME. | RESIDENCE. |
|---|---|---|---|
| W. F. Austin | South Carolina | S. M. Gunter | South Carolina |
| F. R. Brinson | Georgia | W. B. Jackson | Georgia |
| T. K. Bryan | Virginia | J. C. Jefferds | Georgia |
| O. L. Branch | Georgia | M. A. Jefferds | Georgia |
| C. L. Burrows | Georgia | S. A. King | Texas |
| W. B. Cochran | Georgia | F. G. Lieberman | Georgia |
| J. L. Chapman | Texas | B. C. Murray | Georgia |
| M. D. Cowart | Georgia | R. A. Smith | Georgia |
| D. B. Caswell | Georgia | G. G. Stallworth | Alabama |
| S. B. Corn | Georgia | G. H. Tuttle | Alabama |
| J. P. Doster | Georgia | M. H. Tuttle | Alabama |
| H. A. Durant | South Carolina | C. D. Turner | Georgia |
| E. G. Gilder | Texas | J. M. Whitehead | Georgia |

Total, 26.

### Missouri Dental College.

The twenty-eighth annual commencement exercises of the Missouri Dental College, Dental Department of Washington University, St. Louis, Mo., were held in connection with those of the St. Louis Medical College, on Thursday, March 15, 1894.

The number of matriculates for the session was ninety-four.

The degree of D.D.S. was conferred on the following graduates by W. S. Chaplin, A.M., Chancellor of the University:

| NAME. | RESIDENCE. | NAME. | RESIDENCE. |
|---|---|---|---|
| E. F. Becker | Missouri | Dee Morrow | Illinois |
| F. O. Burket | Kansas | G. A. Penney | Illinois |
| O. Conzelman | Missouri | C. W. Purdey | Illinois |
| G. L. Finch | Illinois | M. Robb | Missouri |
| W. F. Hempelman | Missouri | J. Tiffin | Illinois |
| J. B. Kimbrough | Missouri | E. M. Valentine | South Dakota |
| W. J. Lierman | Missouri | J. I. Williams | Illinois |
| R. E. Lockridge | Missouri | M. R. Windhorst | Missouri |
| C. E. McIlwain | Illinois | G. W. Wommack | Missouri |
| H. W. Miles | Nebraska | R. B. Young | Missouri |
| J. S. Morrison | Illinois | | |

Total, 21.

### Western Dental College.

The fourth annual commencement exercises of the Western Dental College were held at the Auditorium, Kansas City, Mo., on Tuesday evening, March 6, 1894.

The annual address was delivered by Rev. C. C. Woods, the address on behalf of the faculty by Dr. Jabez N. Jackson, and the valedictory address by J. J. Moore, D.D.S.

The number of matriculates for the session was one hundred and twenty-six.

The degree of D.D.S. was conferred on the following graduates by Dr. D. J. McMillen, Dean of the College:

| NAME. | RESIDENCE. | NAME. | RESIDENCE. |
|---|---|---|---|
| M. E. Bryan | Missouri | Harry McMillen | Missouri |
| Frank O. Boyd | Kansas | W. Magruder | Missouri |
| R. M. Ball | Missouri | J. J. Moore | Kansas |
| E. E. Buckle | Illinois | H. F. Palenski | Kansas |
| R. M. Bell | Missouri | W. H. Pfahler | Missouri |
| J. B. Douglas | Missouri | H. C. Rush | Kansas |
| E. B. Downing | Missouri | C. A. Stuerwald | Iowa |
| C. P. Fyler | Kansas | B. C. Smith | Missouri |
| J. R. Gaines | Missouri | H. F. Sidney | Kansas |
| E. E. Hawley | Missouri | Lovell Shockey | Missouri |
| J. D. Houston | Nebraska | O. E. Tibbitts | Illinois |
| C. I. Irwin | Missouri | H. C. Thornton | Missouri |
| John C. Kerner | Missouri | Frank Wild | Missouri |

Total, 26.

### University of Denver—Dental Department.

The sixth annual commencement exercises of the Dental Department of the University of Denver were held in connection with those of the Medical and Pharmaceutical Departments, on Tuesday evening, April 17, 1894.

The number of matriculates for the session was eighteen.

Addresses were delivered by Robert McIntyre, Professors Howell T. Pershing, Joseph A. Sewall and J. M. Norman.

The degree of D.D.S. was conferred by Chancellor McDowell on the following graduates:

Orlando Walter Brown.

Herschel David Raub.

Arthur Ward Saner.

Total, 3.

### Vanderbilt University—Dental Department.

The fifteenth annual commencement exercises of the Dental Department of this School were held in the University Chapel, Nashville, Tenn., on Wednesday, February 28, 1894.

The charge to the class was delivered by Professor W. H. Morgan, and the valedictory by A. S. Robertson, of Louisiana.

The number of matriculates for the session was one hundred and twenty-nine.

The degree of D.D.S. was conferred on the following graduates by the Chancellor, J. H. Kirkland:

| NAME. | RESIDENCE. | NAME. | RESIDENCE. |
|---|---|---|---|
| J. S. Betts | South Carolina | G. T. Penny | Missouri |
| A. Blomstine | Tennessee | T. B. Ragin | Illinois |
| W. W. Cross | California | A. S. Robertson | Louisiana |
| T. E. Crosley | Alabama | W. E. Rush | Alabama |
| C. E. Donnell | Missouri | I. F. Scott | Mississippi |
| Wm. W. Earhart | Tennessee | H. M. Stryker | Illinois |
| T. E. Green | Alabama | E. F. Thames | Alabama |
| S. J. Gordon | Alabama | N. N. Vann | Alabama |
| E. O. Hopps | Florida | H. S. Vaughn | Tennessee |
| A. J. Hagen | Tennessee | Wm. W. Walker | Canada |
| J. C. Haines | Illinois | F. W. Wallace | Mississippi |
| R. E. Milburne | Ohio | D. E. Whitby | Alabama |
| L. Leslie | Illinois | | |

Total, 25.

### Illnesses Ascribed to Dentition.

In the course of five years the author saw 734 sick children, and among these 72 who were cutting teeth. In seven cases he believed a direct connection between the illness and the teething could be traced, but in these cases the disturbance was very slight, consisting of restlessness and crying, evidently caused by the pain. Generally the gums were found to be red and swollen. Fever was present in none of the cases.

(This further emphasizes the recent position that severe symptoms during dentition can almost invariably be found to be caused by other disturbances than the mere physiological eruption of the teeth.—Ed.)—*Dr. Sejournet* (*Rev. Mens. des Mal. del'Enfance,* 1893; XI; 3), *in the American Medico-Surgical Bulletin.*

